# American Medical Association

**Published:** 1881-06

**Authors:** 


AMERICAN MEDICAL ASSOCIATION.—We are indebted to Dr. Edwards of the *Virginia Medical Monthly*, for copies of the daily proceedings of that body, during its recent session in Richmond; and from a careful perusal of these reports we are more than ever convinced that the amount of actual benefit to medical science is microscopic in size when contrasted with the amount of actual brain matter present at some of the annual meetings. The reasons for this, however, are easily found in the fact that courtesy and lack of time prevent able men from evolving from the subjects presented for discussion the important truths they contain, or are capable of developing.

Take, for instance, the important subject of blood-letting, referred to in our last issue, and any sane man will accept the dicta of such men as Gross, Davis and Claiborne as their guide in its administration, rather than the captious utterances of lesser lights, who might object without rhyme or reason of their own for the position they assume. Want of time prevents the abler men from discussing,at length the subjects brought before the body; and courtesy compels them to treat with civility, or with silence, the lesser lights around them. These latter get their names in print in this manner, and thus find their way to posterity. There were some engaged in the discussion of blood-letting, who were capable and able to appreciate the vast importance of the remedy, whose opinions were not so pronounced as were those of the distinguished gentlemen we have named, but who were, nevertheless, as strongly convinced of its value as they were; but who courteously permitted others to deliver themselves of *their* views, without interruption. It will ever be thus until the *best* men in *every* community entitled to representation are sent to the meetings of the association, and the time for discussing important subjects prolonged. Socially, the meetings are a success, but in the interests of science, generally failures.

